# Identification and characterization of human nucleus pulposus cell specific serotypes of adeno-associated virus for gene therapeutic approaches of intervertebral disc disorders

**DOI:** 10.1186/s12891-015-0799-4

**Published:** 2015-11-09

**Authors:** Demissew S. Mern, Claudius Thomé

**Affiliations:** Department of Neurosurgery, Innsbruck Medical University, Anichstrasse 35, Innsbruck, A-6020 Austria

**Keywords:** Intervertebral disc disorders, Inflammatory and catabolic cytokines, Nucleus pulposus cells, Nucleus pulposus cell specific adeno-associated virus, Targeted gene therapy, Maintenance of intervertebral disc matrix

## Abstract

**Background:**

Intervertebral disc (IVD) disorders are often accompanied by painful inflammatory and immunopathological processes. Nucleus pulposus (NP) cells play a pivotal role in maintenance of IVD by organizing the expression of anabolic, catabolic, anti-catabolic and inflammatory cytokines. Human NP cells have been targeted by gene therapeutic approaches using lentiviral or adenoviral systems that could be critical due to genome incorporation or immunological side effects. Adeno-associated viruses (AAVs), which do not express any viral gene and are not linked with any known disease in humans, are attractive gene delivery vectors. However, their lack of specific tissue tropism and preexisting immune response are main problems for therapeutic applications. Heretofore, AAVs have not been studied in human IVD research. Therefore, we attempted to identify NP cell specific AAV serotype by targeting human NP cells with different self-complementary AAV (scAAV) serotypes.

Identification and characterization of the proper serotype is crucial to establish less immunogenic and safer gene therapeutic approaches of IVD disorders.

**Methods:**

Preoperative magnetic resonance imaging (MRI) was used for grading of IVD degeneration. NP cells were isolated, cultured with low-glucose and transduced with green fluorescent protein (GFP) packing scAAV serotypes (scAAV1-8) in a dose-dependent manner. scAAV titers were determined by quantitative polymerase chain reaction (qPCR). Transduction efficiencies were determined by fluorescence microscopy and fluorescence-activated cell sorting within 48 days of post-transduction. The 3-(4, 5-dimethylthiazolyl-2)-2,5-diphenyltetrazolium bromide (MTT) assay was used to determine NP cell viability. Three-dimensional (3D) cell culture and enzyme-linked immunosorbant assay (ELISA) were performed to examine the expression levels of inflammatory, catabolic and matrix proteins in NP cells.

**Results:**

scAAV6, scAAV2 and scAAV3 showed high and prolonged transgene GFP expressions with transdution efficiencies of 98.6 %, 91.5 % and 89.6 % respectively (*p* ≤ 0.002). Unlike scAAV6, the serotypes scAAV2 and scAAV3 declined the viability of NP cells by about 25 % and 10 % respectively (*p* ≤ 0.001). Moreover, scAAV6 did not affect the expression of the inflammatory, catabolic and matrix proteins.

**Conclusions:**

As original primary research evaluating AAVs in degenerative human IVDs, this study identified scAAV6 as a proper serotype for high, stable and non-immunogenic target gene expression in human NP cells. The data could be very important to design efficient and safer gene therapeutic approaches of IVD disorders.

## Background

Painful progressive degeneration of IVD, referred to as degenerative disc disorders, has a dramatic influence on the quality of life and causes exceedingly high health care and other socioeconomic expenses [1–4]. Structural failure with accelerated grade of degeneration is usually accompanied by painful inflammatory and patho-immunological processes [5–7]. They are associated with upregulation of pain-mediating inflammatory cytokines, pathological ingrowth of nerves into the inner layers of IVD and progressive spinal instabilities [8–14]. Although current surgical treatment procedures provide relief to the pain and disability, they are not able to restore the biological functions of the IVD. Nevertheless, *in vitro* and in vivo researches of IVD degeneration have identified several therapeutic target genes with significant impact on IVD matrix anabolism and catabolism [15–18]. This provides the opportunity to develop novel gene therapeutic approaches. Gene therapeutic approaches of degenerative discs have been tried *in vitro* and in small animal models through transgenic expression of anabolic factors or inhibition of catabolic or inflammatory cytokines [19–23].

They are usually performed using lentiviral or adenoviral gene delivery systems, which could be critical for future clinical applications due to random gene incorporation and immune reactions [24–33]. An alternative gene delivery system could be the AAV system, which is stable, less immunogenic, non-pathogenic and possibly safer. AAV does not express any viral gene and, as yet, it has not been linked with any known disease in humans [34, 35]. It can have high transduction efficiency in dividing and non-dividing cells and permit prolonged transgene expression of therapeutic genes [34, 35]. In naturally occurring AAV the second strand synthesis is considered to be one of several blocks to efficient infection. However, in the modified form of AAV, known as self-complementary AAV (scAAV), the right inverted terminal repeat (ITR) contains a deletion of D-sequence (the packaging signal) and a terminal resolution site mutation (Δtrs), which prevent Rep mediated nicking and force packaging of dimer or self-complementary genomes [36]. This makes scAAV attractive for gene therapeutic approaches.

There are different AAV serotypes described in the literature that could infect human cells from diverse tissue types with differences in cellular tropism [37]. The use of AAV for targeted gene therapy in human intervertebral disc research is a new approach that has not yet been studied. So far, identification of specific AAV serotypes having human IVD tissue tropism is not attempted.

Here we tried to efficiently target degenerative human NP cells using different scAAV serotypes (scAAV1-8). Preoperative MRI was used for grading of lumbar disc degeneration [38, 39]. Degenerative NP tissues were isolated from patient IVDs of degeneration grade III-V, which were operated due to lumbar disc herniation. NP cells were then immediately isolated from the tissue, cultured with low-glucose medium and transduced with scAAV serotypes in a dose-dependent manner. scAAV titers were determined by qPCR. Transduction efficiencies of of the serotypes were determined by the intensity of transgene GFP expression using fluorescence microscopy and fluorescence-activated cell sorting (FACS) within 48 days. MTT Assay was used to determine the impact of scAAVs on NP cell viability. Three-dimensional cell culture and ELISA were performed to examine the influence of scAAVs on the expression levels of inflammatory, catabolic and matrix proteins in NP cells.

This is the first study to evaluate the serotypes of AAVs in degenerative human IVDs and its findings might contribute to design efficient and possibly safer gene therapeutic approaches of intervertebral disc disorders.

## Methods

### Ethics statement and NP tissue recruitment

Experimental studies with human IVD specimens were approved by the local research ethics committee (Innsbruck Medical University: project AN2014-0027 333/4.24). NP tissues were recruited from patients during lumbar disc surgery with informed consents of the patients. Patients provided their written informed consent to participate in this study. Disc degeneration grade (DDG) was determined by preoperative MRI [38, 39]. NP tissues were recruited from NP compartment during surgical procedure and taken to the laboratory in sterile phosphate buffered saline solution (PBS) (Sigma-Aldrich) for immediate cell isolation. Table 1 exhibits 12 lumbar discs involved in this study.

**Table 1:** Lumbar NP tissue samples obtained from 12 patients undergone surgery due to spinal disc herniation. Table shows details of the NP tissue samples with disc level and disc degeneration grade (DDG)

NP Tissue	Disc level	DDG
1	L5/S1	III
2	L4/L5	III
3	L4/L5	III
4	L4/L5	III
5	L5/S1	IV
6	L4/L5	IV
7	L5/S1	IV
8	L5/S1	IV
9	L5/S1	V
10	L4/L5	V
11	L5/S1	V
12	L5/S1	V

### NP cell isolation and monolayer cell culture

NP tissues were washed in PBS and separated from anulus fibrosus (AF) on the basis of their macroscopic morphology (identification of the innermost lamellar rings of the AF). NP tissues were finely minced into small fragments of approximately 2 mm^3^ and digested with 0.02 % w/v pronase (Sigma-Aldrich) (37 °C, 5 % CO_2_, 1 h) in 20 ml DMEM (Dulbecco's Modified Eagle's Medium), containing 1 % penicillin/streptomycin, 1 % glucose and 10 % FCS (fetal calf serum) (Sigma-Aldrich). After filtration through sterile 75 gm nylon mesh filters (Sigma-Aldrich) and centrifugation of the supernatants (1000 x g, 2 min), pellets were resuspended in 20 ml DMEM and digested with 0.02 % w/v collagenase II (Sigma-Aldrich) and 100U hyaluronidase (Sigma-Aldrich) (37 °C, 5 % CO_2_, 3 h). Following filteration and centrifugation of supernatants, pellets were resuspended in 10 ml DMEM and cultured in 25 cm^2^ tissue culture flask (Sigma-Aldrich) (37 °C, 5 % CO_2_, 2 weeks) by changing the medium every two days. Depending on the amount of cells that could be isolated from the tissues, NP cells could reach 100 % confluentbetween two and three weeks. Cells were cryopreserved at −196 °C in DMEM containing 30 % FCS and 15 % dimethyl sulfoxide (DMSO) (Sigma-Aldrich).

### Helper virus free production of scAAV serotypes

The AAV helper virus free system provides a safer and more convenient gene delivery system. Most of the adenovirus gene products (E2A, E4, and VA RNA genes) required for the production of infective recombinant AAV (rAAV) particles are cloned on the helper plasmid of the respective serotype (pDP1rs, pDP2rs, pDP3rs, pDP4rs, pDP5rs, pDP6rs, and pDP8rs) (PlasmidFactory, Bielefeld, Germany). For vector production of scAAV serotypes, HEK293 cells were cultured in DMEM and passaged 2 times prior to transfection. 5 × 10^6^ cells were grown in 15 cm culture dish of 20 ml culture medium to a confluence of 70 - 80 %. 30 μg of the scAAV shuttle plasmid [40], encoding the expression cassettes of GFP under the control of human CMV promoter, and 96 μg helper plasmids of the respective serotype were used for co-transfection. 30 μg of the scAAV shuttle plasmid and 96 μg of the respective helper plasmid were added in 2.5 ml of 300 mM calcium phosphate (Sigma-Aldrich), gently mixed with 2.5 ml of 2 x HBS (Hepes Buffered Saline) (Sigma-Aldrich) and directly pipetted to the culture dish. Following incubation (37 °C, 5 % CO_2_, 6 h) the transfection medium was changed with prewarmed DMEM containing 2 % FCS. Transfected cells and culture medium were harvested 72 h after transfection and centrifuged (2000 x g, 5 min). Pellet was resuspended in 2.5 ml of serum-free DMEM, treated with benzonase (250 U/ml, 37 °C, 1 h) (Merck Millipore) and subjected to four rounds of freeze/thaw cycles by alternating the tube between the dry ice-ethanol bath and the 37 °C water bath. AAV supernatant was then collected by centrifugation (8000 x g, 30 min) and stored at −80 °C for subsequent purification.

### Purification and quantification of scAAV serotype vectors

Purification of scAAV serotype vectors was performed as previously described [41]. Briefly scAAV vectors were purified from benzonase treated and cleared freeze/thaw-supernatants by iodixanol (Sigma-Aldrich) gradient centrifugation. Iodixanol was then removed by running the iodixanol fractions through PD10 gel filtration columns (GE Healthcare). The eluate was collected in 10 fractions of 1 ml and fractions 4 to 6 were pooled and used for quantification.

Quantitative PCR was applied for quantification of scAAV vector titers using LightCycler 480 (Roche Applied Science) and the TaqMan Gene Expression Master Mix (Life Technologies). PCR reactions were performed in 20 μl of final volume using 1× master mix, supplemented with 100 nM sense, 100 nM antisense primers of GFP and 2 μl of the standard template DNA. The primers GFP-sense:ACGGCGACGTAAACGGCCAC and GPF-antisense:GCGAAGCACTGCACGCCGTA were used. Standard sample and negative control were run in three replicates of a 96 well-plate. Linearized scAAV vector (shuttle plasmid cleaved with *Kas*I) and genomic DNA of scAAV vector (scAAVstd) were used as standard as previously described [42]. The PCR programm had an initial denaturation step at 95 °C for 10 min, 40 cycles of denaturation at 95 °C for 15 s, an extension at 60 °C for 1 min and a melt curve stage (65 °C to 95 °C, increment 0.5 °C). Data analysis was performed using the Applied Biosystems StepOne software v2.1 (Life Technologies).

### Transduction of human NP cells with scAAV serotype vectors

NP cells from 12 lumbar discs of degeneration grade III-V (Table 1) were seeded in 24-well plates at a density of 1 × 10^5^ cells per well (about 50 % confluent) in a volume of 500 μl culture medium containing 1 % FBS. The next day NP cells of each lumbar disc were initially transduced with scAAV1-8 at different doses of vector genome copy per seeded cell (5 vg/c, 50 vg/c, 500 vg/c and 5000 vg/c). After preliminary screening additional transductions were performed with selected serotypes and effective dose to quantify the transduction efficiencies, evaluate potential cell toxicities and determine prolonged transgene expressions.

### Evaluation of transduction efficiencies using fluorescence microscopy and FACS

For preliminary screening of scAAV serotypes having human NP tissue tropism, transduced NP cells were visualized using an AxioVert. A microscope (Carl Zeiss). Fluorescence micrographs were acquired every 2 days for the first 24 days and weekly up to 48 days of post-transduction. Selected scAAV serotypes and viral dose, which efficiently transduced NP cells, were used for further quantification of transduction efficiencies by FACS. For FACS analysis NP cells were seeded as described above and transduced with selected scAAV serotypes and viral dose. Transduced cells were harvested on day 8, 16, 24, 32 and 48, and 1 × 10^5^ cells per sample were counted. The proportion of GFP-positive cells was determined using MoFlo cell sorter (Beckman Coulter) according to the manufacturer’s protocol. Briefly, a MoFlo cell sorter with a 100-mm flow cell tip and a flow rate of 12000 events per second, with an extention wavelength of 488 nm and a laser power of 110 W was used.

### Cell viability assay

To analyse the cytotoxic effects of scAAV serotypes, which are able to effectively transduce human NP cells, the number of viable NP cells were quantified using 3-(4, 5-dimethylthiazolyl-2)-2,5-diphenyltetrazolium bromide (MTT Assay Kit, Molecular Probes). Transduced and non-transduced (negative control) NP cells were harvested after 8, 16, 24, 32 and 48 days of post-transduction. NP cells were trypsinated, centrifugated and pellets were washed twice with 1 x PBS and resuspended in 250 μl culture medium. Duplicates of 100 μl suspended cells were plated into flat-bottomed 96-well plate. Duplicate control wells of medium alone were added to provide the blanks for absorbance readings. After incubation for recovering (37 °C, 5 % CO_2_, 24 h), 10 μl MTT reagent was added to each well and incubated again for 3 h. Then 100 μl of the SDS-HCl (Sodium dodecyl sulfate - hydrochloric acid) solution was added to each well for further incubation of 4 h. The absorbance was measured at 570 nm in a microtiter plate reader Infinite 200 (TECAN) and the average value of the blank duplicate readings was subtracted from the average values of the sample duplicate readings. Number of viable NP cells was calculated from the standard curve.

### Three-dimensional NP cell culture

For three-dimensional culture (3D) of NP cells the collagen type I scaffold of 24-well plate format was used (Viscofan Bioengineering). 250 μl PBS (pH 7.3 without Ca2^+^/Mg2^+^) was added to each well for attaching of the scaffold to the bottom of the well. The scaffold was taken out of the blister with sterilized forceps, placed onto the PBS without submerging and incubated at room temperature for 20 min. After removing of the remaining PBS, the plate was left in the operating laminar flow hood overnight. The scaffold was equilibrated with 250 ml pre-warmed culture medium (37 °C, 5 % CO_2_, 10 min) and monolayer scAAV transduced NP cells were transferred to collagen I scaffold on day 8 of posttransduction. NP cells were cultured in 500 μl culture medium (37 °C, 5 % CO2) by changing the culture medium every two days. As required, NP cells were harvested by digestion of the scaffold with 0.02 % w/v collagenase II in 250 μl medium (37 °C, 5 % CO_2_, 1 h). Cell suspension was filtered through sterile 75 gm nylon mesh filter and supernatant was centrifuged (1000 x g, 2 min). Then pellet was washed twice in 1 ml PBS (1000 x g, 2 min) and processed for assays of inflammatory, catabolic and matrix proteins expressions.

### Enzyme-linked immunosorbant assay of inflammatory, catabolic and matrix proteins

To examine the impact of scAAV vectors on the expression levels of inflammatory, catabolic and matrix proteins in NP cells, ELISA was performed on 100 μg of total protein extracts from each sample for each independent experiment. As described above, NP cells were seeded and transduced with the favoured scAAV serotype at its effective dose. On day 8 of post-transduction NP cells were harvested and cell pellet was washed with 1 x PBS, resuspended in 500 μl culture medium and three-dimensional cultured for 30 days. Cells were harvested by digestion of the scaffold as described above and for protein isolation pellet was washed twice in cold 1 x PBS (2500 x g, 5 min) and resuspended with 100 μl cold radio-immunoprecipitation assay (RIPA) buffer (Sigma-Aldrich) containing protease and phosphatase inhibitor cocktails (Sigma-Aldrich). After sonication at 50 % pulse for 30 s, the mixture was shacked gently on ice (15 min), centrifuged (14000 x g, 4 °C and 15 min) and supernatant was transferred to new tube for protein quantification. Protein concentration in sample was determined using BCA Protein Assay Kit (Thermo Scientific) according to the instruction manual. For measuring the concentration of inflammatory, catabolic and matrix proteins in NP cells ELISA kits were used (R&D Systems, Uscn Life Science Inc). Assays were performed according to the instruction manuals. The analysed target proteins include: the inflammatory cytokines IL-1β (interleukin-1β) and TNF-α (tumornekrosefaktor-α); the catabolic factors ADAMTS-4, ADAMTS-5 (a disintegrin and metalloproteinase with thrombospondin motifs) as well as matrix proteins aggrecan and collagen type II.

### Statistical data analysis

To estimate the reliability of the MRI evaluations of IVD degeneration, Landis and Koch based interpretations with κ statistics and agreement percentage among two observers (interobserver reliability) were used [38, 39]. The software IBM SPSS Statistics 20, Armonk New York USA was used for statistical analysis. 1-way ANOVA and pairwise comparisons were used to compare scAAV treated and non-treated cells. Significance was set at *P* < 0.05.

## Results

### Reliability of MRI grading

The interobserver reliability agreement between two observers, for the MRI rating of lumbar disc degeneration grade, was perfect with 100 % frequency of agreement and κ = 1.00.

### Production of adenovirus free high-titer scAAV serotype vectors

As described in materials and methods, scAAV serotype vectors were produced, purified and quantified using qPCR. High titers of scAAV vectors were verified for all serotypes. Final titers between 3 × 10^11^ and 1.8 × 10^12^ vector genome copies were determined from 3 × 10^7^ HEK293 cells.

### Transduction efficiency of scAAV serotypes in NP cells

**Fig. 1:**
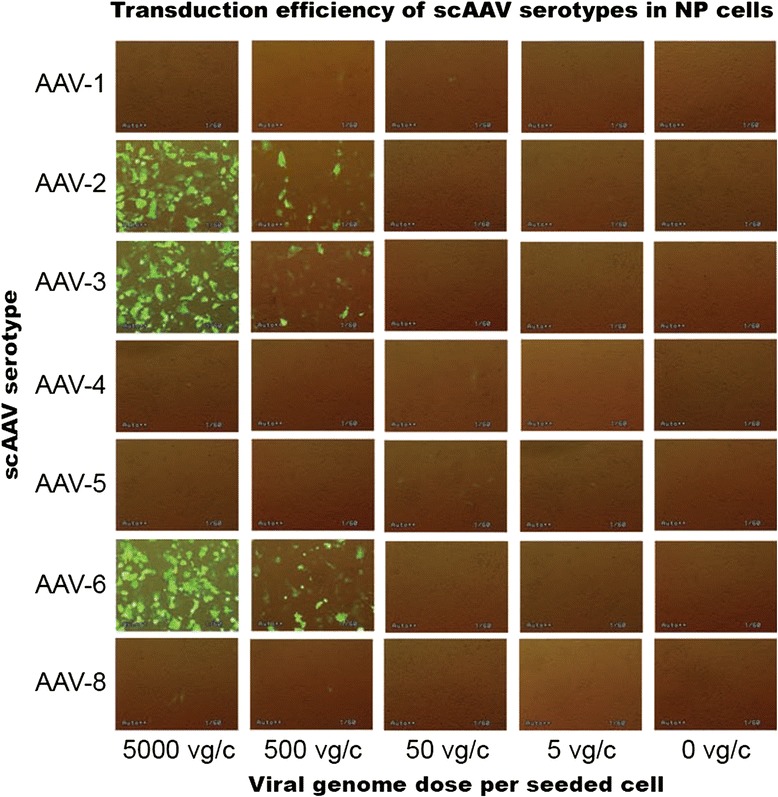
Fluorescence micrographs of the initial scAAV serotype screening on day 8 of post-transduction. For the primary screening of the serotypes in NP cells, 1 x 10^5^ NP cells were seeded and transduced with GFP packing scAAV serotypes at different viral genome copies per seeded cell (vg/c) and fluorescence micrographs were acquired up to 48 days. On day 8 of post-transduction scAAV2, scAAV3 and scAAV6 displayed the highest transduction efficiencies at a dose of 5000 vg/c. Original magnification: x 40

For the early screening of scAAV serotypes that may have NP tissue tropism, NP cells from 12 lumbar discs of degeneration grade III-V (Table 1) were transduced with GFP packing scAAV serotypes at different viral doses and fluorescence micrographs were acquired up to 48 days as described in materials and methods. The initial screening showed that the serotypes scAAV2, scAAV3 and scAAV6 at the dose of 5000 vg/c could transduce NP cells with highest efficiencies, as shown by fluorescence micrographs from day 8 of post-transduction (Fig. 1). The serotypes scAAV1, scAAV4, scAAV5 and scAAV8 did not show any GFP expression at all examined time and viral doses.

**Fig. 2:**
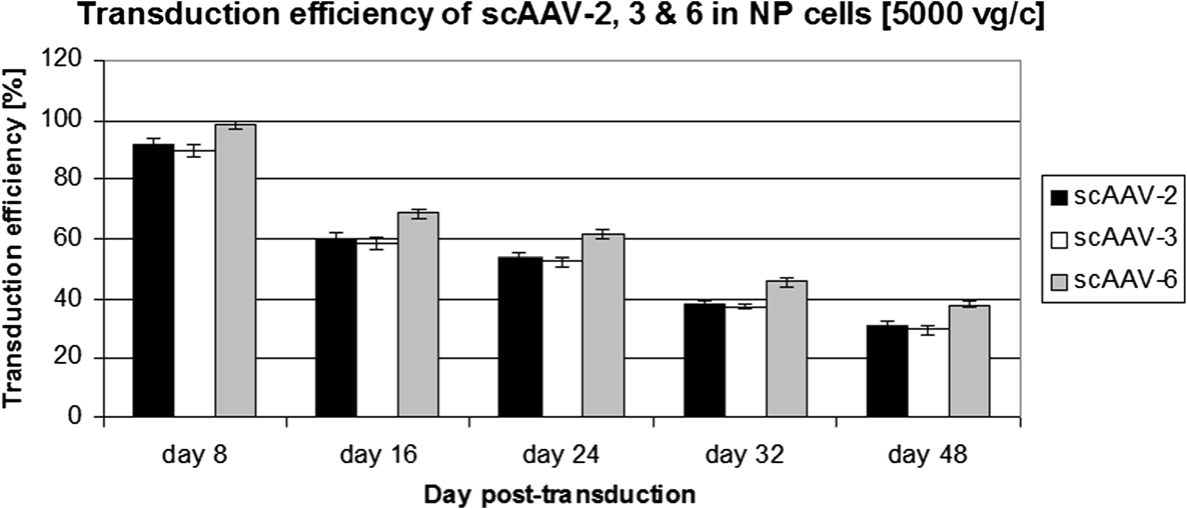
FACS based transduction efficiencies of scAAV2, scAAV3 and scAAV6 in NP cells. 1 x 10^5^ NP cells were seeded and after transduction at a viral dose of 5000 vg/c, GFP positive cells were quantified by FACS on day 8, 16, 24, 32 and 48 of post-transduction. Data represent the mean with standard deviation (SD) of three independent experiments, each performed in triplicate (*p* < 0.05). Error bars indicate SD values

Based on the initial screening the serotypes scAAV2, scAAV3 and scAAV6 and the viral dose 5000 vg/c were selected for quantitative analysis of their transduction efficiencies by FACS. As described above, transduced NP cells were harvested on day 8, 16, 24, 32 and 48 of post-transduction and 1 × 10^5^ cells per sample were counted to determine the proportion of GFP-positive cells. On day 8 of post-transduction an average of 98.6 % GFP positive cells were detected for scAAV6 with minimum and maximum transduction values between 92.8 % and 99.9 % (Table 2 and Fig. 2). Lower transduction efficiencies of 91.6 % and 89.6 % were detected for scAAV2 and scAAV3 with minimum and maximum transduction values of (87.2 % & 95.4 %) and (85.0 % & 92.9 %) respectively (*p* ≤ 0.002) (Table 2 and Fig. 2). Analysis of samples on day 16 exhibited lower transduction efficiencies of 68.5 %, 60.3 % and 58.6 % for scAAV6, scAAV2 and scAAV3 with minimum and maximum transduction values of (66.1 % & 71.5 %), (55.8 % & 65.9 %) and (54.8 % & 60 %) respectively (*p* ≤ 0.005). On day 24, 32 and 48 decreasing transduction efficiencies of (61.5 %, 45.7 % & 38 %) for scAAV6, (53.6 %, 38.2 % & 31.2 %) for scAAV2 and (52.3 %, 37.3 % & 29.4 %) for scAAV3 were detected (*p* ≤ 0.003). On all days of GFP detection scAAV6 continuously displayed the highest transduction efficiencies, whereas the transduction efficiencies scAAV2 and scAAV3 were comparable. The serotypes scAAV6, scAAV2 and scAAV3 at transduction dose of 5000 vg/c demonstrated long-term transgene expressions in NP cells. Degeneration grades of IVDs did not show any influence on the transduction efficiencies of the analysed serotypes.

**Table 2:** GFP based quantification of transduction efficiencies by FACS in NP cells transduced with scAAV serotypes

FACS day	Serotype	Min. value %	Max. value %	Range %	Mean value %	SD %
day 8	scAAV2	87.2	95.4	8.2	91.5	1.943
	scAAV3	85.0	92.9	7.8	89.6	1.866
	scAAV6	92.8	99.9	7.1	98.6	1.493
day 16	scAAV2	55.8	65.9	10.1	60.4	1.876
	scAAV3	54.8	62.0	7.1	58.5	1.853
	scAAV6	66.1	71.5	5.3	68.5	1.474
day 24	scAAV2	50.3	56.9	6.5	53.6	1.677
	scAAV3	48.0	54.8	6.8	52.3	1.623
	scAAV6	57.3	64.3	7.0	61.5	1.755
day 32	scAAV2	35.9	40.6	4.6	38.2	1.137
	scAAV3	34.9	39.9	5.0	37.3	1.256
	scAAV6	41.8	49.1	7.2	45.7	1.669
day 48	scAAV2	29.0	33.3	4.3	31.2	1.242
	scAAV3	26.2	31.5	5.3	29.3	1.461
	scAAV6	35.8	40.7	4.8	38.0	1.196

1 x 10^5^ NP cells were seeded and transduced with scAAV2, scAAV3 and scAAV6 at viral dose of 5000 vg/c. FACS was done on day 8, 16, 24, 32 and 48 of post-transduction. Min and Max values represent the minimum and maximum transduction efficiencies measured throughout the NP samples. Range represents the fluctuation between maximum and minimum values. Data represent the mean with standard deviation (SD) of three independent experiments, each performed in triplicate (*p* < 0.05)

### Effect of scAAV2, scAAV3 and scAAV6 on the viability of NP cells

**Fig. 3:**
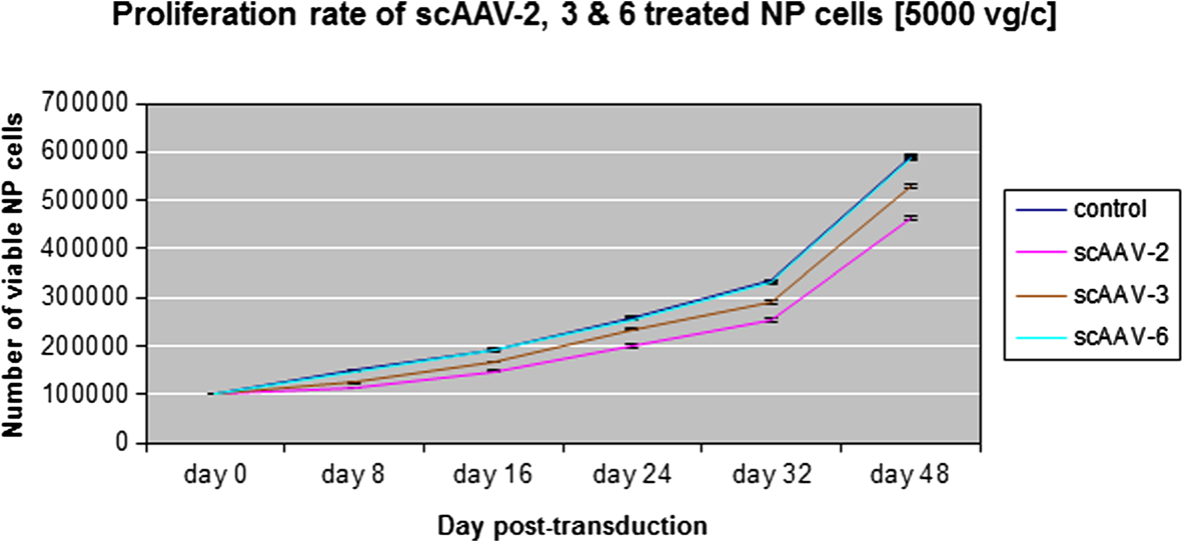
NP cell viabilities after transduction with scAAV2, scAAV3 and scAAV6. A viral dose of 5000 vg/c was used to transduce 1 x 10^5^ NP cells with scAAV2, scAAV3 and scAAV6. After transduction MTT assays were done on day 8, 16, 24, 32 and 48 of post-transduction to quantify the number of viable cells. Data represent the mean number of viable cells with standard deviation (SD) of three independent experiments, each performed in duplicat (*p* < 0.005). Error bars indicate SD values

**Fig. 4:**
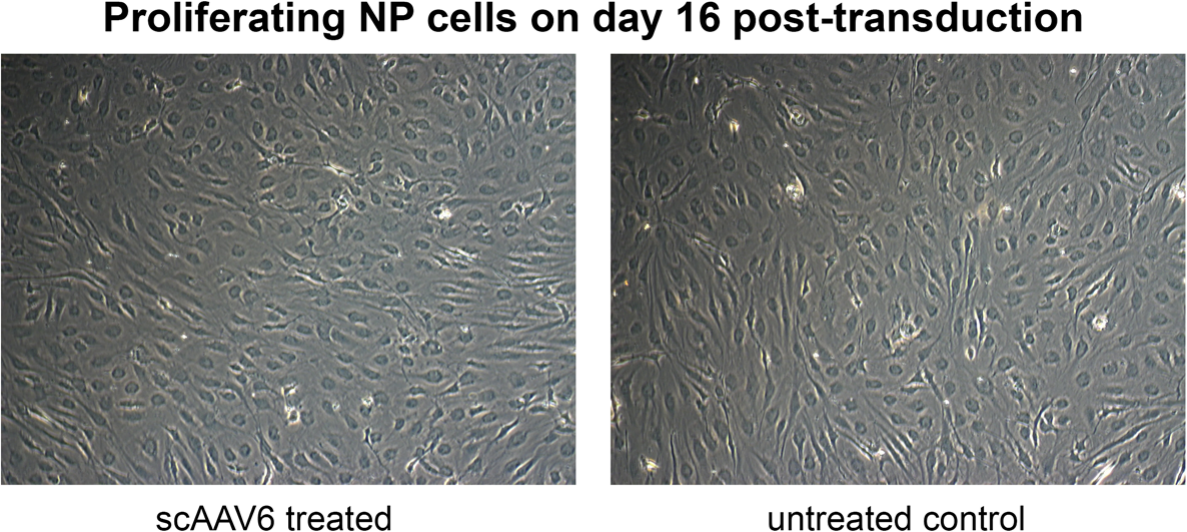
Proliferating NP cells on day 16 after transduction with scAAV6. A viral dose of 5000 vg/c was used to transduce 1 x 10^5^ NP cells with scAAV6 at 50 % confluent and about 95 % viabilily. Images show bright field views of scAAV6 treated and untreated control NP cells on day 16 of post-transduction. Morphological chages were not observed as shown as an example herein. Original magnification: x 20

To examine the potential impact of scAAV2, scAAV3 and scAAV6 on NP cell viability 1 × 10^5^ NP cells were transduced with scAAV2, scAAV3 and scAAV6 at a viral dose of 5000 vg/c. MTT assays were done on day 8, 16, 24, 32 and 48 of post-transduction to quantify the number of viable NP cells. All through the days of MTT assays, similar viabilities of scAAV6 treated and untreated cells (negative control) were recorded with mean number of viable cells (145643 ± 1411 and 149148 ± 1009) for day 8 and (586593 ± 4967 and 589681 ± 3362) for day 48 respectively (*p* < 0.081) (Table 3 and Fig. 3). No morphological changes were observed between scAAV6 treated and untreated NP cells, as shown as an example in Fig. 4. The viability of scAAV2 treated cells was reduced by about 25 % with mean number of viable cells (113127 ± 1668) and (463623 ± 3952) for day 8 and 48 respectively (*p* < 0.001). Although the viability of scAAV3 treated cells was better than that of the scAAV2 treated cells, their viability, related to that of the scAAV6 treated cells, was reduced by more than 10 % with mean number of viable cells (123754 ± 1406) and (529090 ± 4556) for day 8 and 48 respectively (*p* < 0.001) (Table 3 and Fig. 3). Degeneration grades of IVDs did not show any influence on the proliferation of NP cells.

**Table 3:** Viability of NP cells transduced with scAAV2, scAAV3 and scAAV6

Day post-transduction		day 8	day 16	day 24	day 32	day 48
Control	Mean	149148	192663	258666	334200	589681
	SD	1009	1027	3446	2628	3362
scAAV2	Mean	113127	146759	198713	252733	463623
	SD	1668	1863	3214	3745	3952
scAAV3	Mean	123754	166629	233436	291172	529090
	SD	1406	1541	1878	4370	4556
scAAV6	Mean	145643	189679	254178	330270	586593
	SD	1411	1540	2977	4966	4967

1 x 10^5^ NP cells were transduced scAAV2, scAAV3 and scAAV6 at a viral dose of 5000 vg/c. On day 8, 16, 24, 32 and 48 of post-transduction MTT assays were performed to measure the number of viable cells. Data represent the mean number of viable cells with standard deviation (SD) of three independent experiments, each performed in duplicate (*p* < 0.05)

### Levels of inflammatory, catabolic and matrix proteins in scAAV6 treated NP cells

**Fig. 5:**
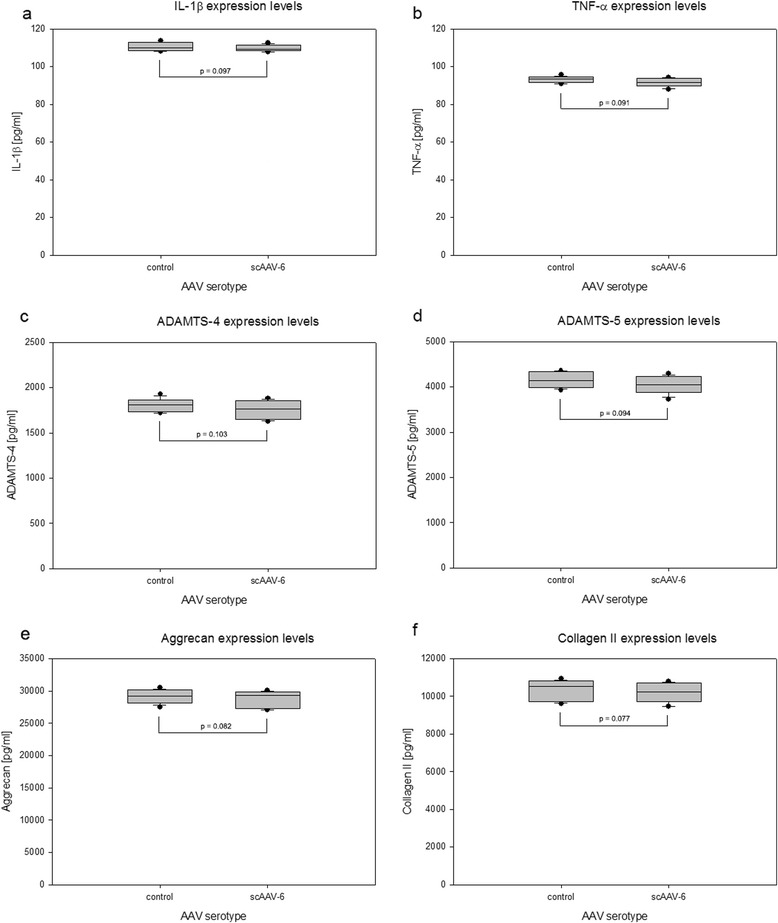
scAAV6 treated NP cells and their expression levels of inflammatory, catabolic and matrix proteins. 1 x 10^5^ NP cells from grade III samples were monolayer transduced with scAAV6 [5000 vg/c] and 3D cultured for four weeks in collagen I scaffold. To determine expression levels of inflammatory, catabolic and matrix proteins [pg/ml], ELISA assays were performed using 100 μg of total protein extracts from each sample. Box plots with whiskers min. to max. show the expression levels of the inflammatory cytokines IL-1β and TNF-α (**a**, **b**), the catabolic factors ADAMTS-4 and ADAMTS-5 (**c**, **d**) and the IVD matrix proteins aggrecan and collagen II (**e**, **f**). Data were obtained from three independent experiments, each performed in duplicate (*p* < 0.05)

The serotype scAAV6, showing the highest transduction efficiency and no impact on viability of NP cells, can be selected as the hot favourite serotype, if it does not promote the expression of unfavourable inflammatory and catabolic proteins in NP cells. For protein expression analysis monolayer transduced NP cells (scAAV6, 5000 vg/c) were transferred to collagen I scaffold on day 8 of posttransduction and 3D cultured for four weeks. The expression levels of inflammatory cytokines (IL-1β & TNF-α), catabolic factors (ADAMTS-4 & ADAMTS-5) and matrix proteins (aggrecan & collagen II) were determined by using ELISA-assays applied on 100 μg of total protein extracts of each sample. In all samples scAAV6 had shown no impact on the endogeneous expression levels of the inflammatory cytokines, catabolic factors and matrix proteins. Figure 5 and Table 4 exhibit the levels of protein expressions in NP cells of disc degeneration grade III. Comparable protein expression levels [pg/ml] of the inflammatory cytokines were determined in untreated and scAAV6 treated samples with mean expression levels for IL-1β (110 ± 2.247 and 109 ± 1.702) and TNF-α (93 ± 1.648 and 91 ± 2.314) respectively (*p* ≤ 0.097). Similarly, the expression levels of the catabolic factors in untreated and scAAV6 treated samples were equivalent with mean expression levels [pg/ml] for ADAMTS-4 (1807 ± 79.49 and 1756 ± 105.6) and ADAMTS-5 (4156 ± 177.9 and 4042 ± 203.5) respectively (*p* ≤ 0.103). Moreover, the expression levels [pg/ml] of the matrix proteins in untreated and treated samples were similar with mean expression values for aggrecan (29100 ± 111 and 28804 ± 1279) and collagen II (10311 ± 542.7 and 10171 ± 548.9) respectively (*p* ≤ 0.082). Furthermore, the expression levels of the inflammatory, catabolic and matrix proteins were similar in untreated and scAAV6 treated samples of disc degeneration grade IV and V (data not shown).

**Table 4:** Levels of inflammatory, catabolic and matrix proteins in scAAV6 treated NP cells of DDG III

Target protein	NP cell sample	Mean [pg/ml]	SD	Mean fold
IL-1β	control	110	2.247	1.009
	scAAV6	109	1.702	
TNF-α	control	93	1.648	1.021
	scAAV6	91	2.314	
ADAMTS-4	control	1807	79.49	1.029
	scAAV6	1756	105.6	
ADAMTS-5	control	4156	177.9	1.028
	scAAV6	4042	203.5	
Aggrecan	control	29100	1112	1.010
	scAAV6	28804	1279	
Collagen II	control	10311	542.7	1.013
	scAAV6	10171	548.9	

1 x 10^5^ NP cells were monolayer transduced with scAAV6 [5000 vg/c] and 3D cultured for four weeks in collagen I scaffold. Expression levels [pg/ml] of the inflammatory cytokines (IL-1β & TNF-α), catabolic factors (ADAMTS-4 & ADAMTS-5) and matrix proteins (Aggrecan & Collagen II) were determined using ELISA-assays applied on 100 μg of total protein extracts of each sample. Data represent the mean and standard deviation (SD) of three independent experiments, each performed in duplicate (p < 0.05). The mean fold represents the ratio of the untreated (control) expression level to scAAV6 treated expression level

## Discussion

The regenerative potential of gene therapeutic approaches in IVD cells has been shown in different publications. These include inhibition of catabolic and inflammatory cytokines or activation anabolic factors [13, 16, 43–46]. However, gene therapeutic approaches in degenerative human disc cells have usually been performed using lentiviral or adenoviral gene delivery systems that could be critical for future clinical applications due to their immunological side effects [24–30]. The genome-incorporating lentiviral gene delivery system can randomly incorporate the exogenous therapeutic gene in the host cell genome, which could inactivate crucial housekeeping or tumor suppressor genes [24, 25]. Although the non-genome-incorporating adenoviral gene delivery systems can be used for high levels and persistent expressions of therapeutic genes, their therapeutic potentials are limited by the immune reactions to viral proteins [26–30]. Their applications in spinal discs near to sensitive neural structure could induce toxicity and immunological side-effects, which could result in neurological deficits and serious pain [31–33, 44, 47, 48]. Therefore, the adeno-associated viral gene delivery system, which does not express any viral protein and is not linked with any known disease in humans, has become an interesting therapeutic gene delivery system. Several studies concerning the potential applications of AAV for gene therrapeutic approaches of degenerative discs have been published [49–54]. AAV2 mediated *in vitro* co-transfection of bone morphogenetic protein 7 (BMP-7) and transcription factor SOX-9 has been shown to promote the expression of collagen II in degenerative human NP cells [49]. Similarly AAV2 mediated in vivo co-transfection of BMP-7 and SOX-9 could enhance the expression of collagen II and proteoglycan, and significantly improve the height of degenerative rabbit discs [50]. Furthermore, AAV2 mediated *in vitro* co-transfection of vascular endothelial growth factor165 (hVEGF165) and transforming growth factor-β1 (TGF-β1) has been presented to increase the synthesis of collagen I in degenerative anulus fibrosis cells of rabbit [51]. However in preimmunized rabbits, due to humoral immune response against the AAV vector, decreased transgene expressions have been described [52]. AAV2 based *in vitro* and in vivo testing of gene expression in rabbit IVD has demonstrated the potential of AAV system to improve safety without nonspecific gene expression or expression in adjacent tissues [53]. Adenoviral mediated delivery of anabolic cytokines into the lumbar epidural space of rabbits has demonstrated significant clinical, biochemical, and histologic morbidit by up to 80 % of the rabbits. Conversely, AAV mediated delivery of any anabolic cytokin resulted in no clinical, histologic, or biochemical morbidity [54]. AAV can have high transduction efficiency and permits prolonged expression of therapeutic gene [34, 35]. The self-complementary AAV (scAAV) containing a terminal resolution site mutation and a deletion of D-sequence in the right ITR can prevent Rep mediated nicking and permit efficient infection as well as improved transgene expression [36, 55]. Despite great advantages of using AAVs as vectors, there are some limitations due to their small genome size (4.7 kbp), as large genes are not convenient for cloning in AAV vectors. Moreover, the cloning capacity of scAAVs is even less than the already limited cloning capacity of standard AAVs. However, while a standard AAV packages a single strand DNA and must wait for the complementary strand to be synthesized, scAAV packages two shorter strands that are complementary to each other. By avoiding complementary strand synthesis, scAAV can more quickly express therapeutic target genes. Nevertheless, standard AAV and scAAV vectors have been widely used for the delivery of small therapeutic genes (up to 3.0 kbp) and shRNA expression cassettes in RNAi approaches. Options are currently being explored to overrid this limiting capacity. For instance the ITRs of two AAV genomes can anneal to form head to tail concatamers, which could almost double the coding capacity of the vector.

In human several AAV serotypes with differences in tissue tropism have been described [37]. AAV serotypes regarding human IVD have not been studied. No research has been performed to identify and characterize human IVD specific AAV serotypes. Their transduction efficiency and cell cytotoxicity have not yet been evaluated in human IVD cells. Therefore, we attempted to effectively target human NP cells using different scAAV serotypes and evaluated their transduction efficiency, cell cytotoxicity and effect on the expression of inflammatory, catabolic and matrix proteins. The initial screening of the scAAV serotypes showed that only the serotypes scAAV2, scAAV3 and scAAV6 could transduce NP cells and their highest transduction efficiencies were obtained at the dose of 5000 vg/c by day 8 (Fig. 1). Consequently, the three serotypes [5000 vg/c] were selected to be used for quantitativ analysis of GFP positive cells by FACS. Compared to scAAV2 and scAAV3, the setotype scAAV6 steadily showed the highest transduction efficiencies in all days of GFP positive cell detection (Table 2 and Fig. 2). However, for all serotypes the number of GFP positive cells was gradually decreased from day 8 to 48: from 98.6 % to 38 % for scAAV6, from 91.6 % to 31.2 % for scAAV2 and from 89.6 % to 29.4 % for scAAV3. These continuous reductions in number of GFP positive cells could be due to the fact that NP cells continually devide and as a result of which the AAV vector genome got diluted over time in the growing cell population. The dilution of the AAV vector genome was in line with our expectation, as AAV episome could be lost during cell division [34, 35, 56]. Nevertheless, the recorded long-term transgen expressions are promissing for gene therapeutic approaches of degenerative IVDs, which usually need prolonged regeneration time (weeks to monthes). To investigate the impact of the three scAAV serotypes on NP cell viability, MTT assays were performed. These lower viabilities of scAAV2 and scAAV3 treated NP cells (Table 3 and Fig. 3) might be caused by the immune respons of NP cells to the high dose [5000 vg/c] of scAAV2 and scAAV3, which feature about 88 % sequence homology [57]. Despite the fact that the transduction of NP cells with lower dose of scAAV2 and scAAV3 [3000 vg/c] improved the cell viabilities by 9 % and 12 %, the maximum transduction efficiencies remained below 60 % (data not shown).

Increased protein levels of inflammatory cytokines (IL-1β, TNF-α) and catabolic factors (ADAMTS-4, ADAMTS-5) are considerd to be solid indicators of inflammatory catabolic changes in intervertebral discs [16–18, 58–60]. Increased catabolism in NP tissue, mediated by the inflammatory cytokines and the catabolic factors, reduces the levels the extracellular matrix proteins (aggrecan and collagen II) [16–18]. Since scAAV6 displayed the highest transduction efficency and no impact on NP cell viability, it was chosen for the detection of inflammatory catabolic responses. Using ELISA assays comparable protein expression levels were detected in untreated and scAAV6 treated NP cells (Table 4 and Fig. 5), indicating no inflammatory catabolic responses of NP cells to scAAV6.

## Conclusions

This is the first study to identify, characterize and evaluate AAV serotypes in degenerative human IVDs. Here we identified scAAV2, scAAV3 and scAAV6, which could efficiently transduce human NP cells. scAAV6 seems to be the most convenient for gene therapeutic approaches of degenerative IVDs, as it showed the highest transduction efficiency without any effect on NP cell viability, and did not show any impact on the expression of inflammatory, catabolic and matrix proteins. This study might help to design efficient, stable and less immunogenic gene therapeutic approaches to impede degeneration of spinal discs.

## Abbreviations

AAV: Adeno-associated virus
ADAMTS: A disintegrin and metalloproteinase with thrombospondin motifs
AF: Anulus fibrosus
BCA: Bicinchoninic acid
BMP-7: Bone morphogenetic protein 7
CMV: Cytomegalovirus
DDG: Disc degeneration grade
DMEM: Dulbecco's modified Eagle's medium
DMSO: Dimethyl sulfoxide
ELISA: Enzyme-linked immunosorbant assay
FACS: Fluorescence-activated cell sorting
FCS: Fetal calf serum
GFP: Green fluorescent protein
HBS: Hepes buffered saline
HCl: Hydrochloric acid
HEK293: Human embryonic kidney 293
hVEGF165: vascular endothelial growth factor165
IL-1β: Interleukin-1beta
ITR: Inverted terminal repeat
IVD: Intervertebral disc
MRI: Magnetic resonance imaging
MTT: 3-(4, 5-dimethylthiazolyl-2)-2,5-diphenyltetrazolium bromide
NP: Nucleus pulposus
PBS: Phosphate buffered saline
qPCR: quantitative polymerase chain reaction
rAAV: Recombinant adeno-associated virus
RIPA: Radio-immunoprecipitation assay
scAAV: self-complementary adeno-associated virus
SDS: Sodium dodecyl sulfate
TGF-β1: Transforming growth factor-β1
TNF-α: Tumornekrosefaktor-alfa
3D: three-dimensional
